# A study on monthly sales forecasting of new energy vehicles in urban areas using the WOA-BiGRU model

**DOI:** 10.1371/journal.pone.0320962

**Published:** 2025-04-21

**Authors:** Xiangtu Li

**Affiliations:** School of Artificial Intelligence, Southeast University, Nanjing, China; Federal University of Petroleum Resources Effurun, NIGERIA

## Abstract

To accurately predict the sales of new energy vehicles (NEVs) in Chinese cities and explore the applicability of optimization algorithms for GRU models in forecasting urban NEV sales., this paper conducts a spatiotemporal analysis of urban NEV sales data. The Whale Optimization Algorithm (WOA) is then employed to optimize the parameters of the Bidirectional Gated Recurrent Unit (BiGRU) model, thereby proposing a WOA-BiGRU-based model for monthly sales prediction for urban NEVs. Its prediction results are compared with those of the particle swarm optimization (PSO) algorithm. The research findings are as follows: The growth of NEV sales has reversed the declining trend of overall automobile sales in China; Cities with higher NEV sales are predominantly concentrated in four major economic hubs--the Pearl River Delta, Yangtze River Delta, Beijing-Tianjin-Hebei region, and Chengdu-Chongqing. Optimization techniques such as WOA can improve the accuracy of GRU models in predicting city-level sales of NEV. The WOA-BiGRU model outperforms both the standalone BiGRU and PSO models, achieving a Mean Absolute Error (MAE) of 3051.89, which is 526.18 lower than the BiGRU model and 104.72 lower than that of the PSO model. This study improves the accuracy of monthly sales prediction for urban NEVs, offering critical insights for the development of the NEV industry in China, the deployment of charging infrastructure, the stabilization of the power grid, and emission reduction in the transportation sector.

## Introduction

he electric vehicle (EV) market is the most dynamic segment within the renewable energy sector. According to the *Global EV Outlook 2024* report published by the International Energy Agency (IEA), global EV sales exceeded 14 million units. From 2 million units in 2018, global EV sales surged to over 14 million in 2023, with their market share increasing from 2.5% to 16% [1]. China significantly leads in clean energy technologies, particularly in EVs. In 2023, China’s new energy vehicle (NEV) sales reached 9.495 million units, accounting for 64.8% of global sales. In 2024, the production of NEVs in China reached 12.888 million units, accounting for 40.9% of the country’s total car sales. Since July 2024, the retail penetration rate of NEVs in the Chinese market has exceeded 50% for five consecutive months, with 118 cities surpassing a 50% NEV adoption rate in July alone. NEVs have made tremendous contributions to reducing transportation-related carbon emissions in China, but they have also posed substantial challenges to the layout of charging infrastructure and the stability of the power grid. As the largest EV sales market, accurate forecasting of EV sales in China is of great significance for the development of China’s new energy vehicle industry, the deployment of charging stations the prediction of electricity demand, the stability of the power grid, and the realization of the dual-carbon (carbon peaking and carbon neutrality) goals in the transportation sector.

Currently, the earliest available data on China’s NEV sales dates back to 2008, while city-level sales data begins in 2021. The time span of the collected data is relatively short, and NEV sales are influenced by various factors such as policy, economy, population, temperature, and technology [2]. Both annual and city-level sales of new energy vehicles in China have undergone significant changes. Consequently, the factors considered in building prediction models have evolved from univariate to multivariate. For example, Ning et al. explored the relevant factors affecting NEV sales from both temporal and spatial perspectives [3]; Dai et al. predicted China’s NEV sales data based on univariate (monthly sales data of vehicles) and multivariate (economic, social, and technological) factors, respectively [4]; and Liu constructed GRA-BiLSTM and DWT-BiLSTM prediction models for NEV sales, incorporating multiple factors [5, 6].The methods for predicting NEV sales have evolved, ranging from regression models, power exponential models, to grey prediction, time series models, Bass models, and beyond.. For example, Ma et al. predicted China’s NEV sales using the Analytic Hierarchy Process (AHP) and a Logit regression model [7]; Liu et al. utilized a discrete-time grey power model to forecast China’s NEV sales [8]; Ning et al. employed a multivariate prediction approach combining the Autoregressive Integrated Moving Average (ARIMA) model, its optimized version, and the Gradient Boosting Decision Tree (GBDT) model to predict China’s NEV sales [3]; Qian et al. proposed a grey Lotka-Volterra model based on the HP filter to analyze NEV sales trends [9]; Liu et al. introduced an optimized discrete grey power model to predict the development trend of China’s NEVs [5]; Pei et al. conducted quarterly sales forecasts for China’s NEV industry using a data grouping method based on the nonlinear grey Bernoulli model [10]; Ding et al. proposed a new adaptive optimized grey model for predicting EV sales and inventory, alongside a composite prediction framework for adaptive data preprocessing and optimizing the nonlinear grey Bernoulli model for NEV sales [11–12]; He et al. presented an optimized grey buffer operator for predicting the production and sales of NEVs in China [13]; Sun et al. used an improved Bass model based on product value to predict the sales of new energy passenger vehicles [14]; Bian et al. predicted the demand for NEVs through an enhanced Bass model [15].

With the surge of machine learning, predictions based on models such as BP Neural Networks, Recurrent Neural Networks (RNNs), Convolutional Neural Networks (CNNs), and artificial intelligence models have become research hotspots. For instance: Wang et al. utilized BP Neural Networks to predict NEV sales [16]; Ding et al. constructed a combined model incorporating Backpropagation Neural Networks (BPNN), Recurrent Neural Networks (RNN), and Long Short-Term Memory (LSTM) neural networks to forecast BYD electric vehicle sales [17]; Li et al. proposed a hybrid neural network model based on Convolutional Neural Networks (CNN) and Long Short-Term Memory (LSTM) for accurate prediction of NEV sales [18]; Liu et al. introduced a multi-factor NEV model integrating Discrete Wavelet Transform (DWT) and Bidirectional Long Short-Term Memory (BiLSTM) to predict the market penetration rate of NEVs [6]. Zeng et al. proposed a sales forecasting method for NEVs in China based on a novel intelligent buffering operator [19]. Optimization methods are fundamental to model training in machine learning, as they identify the most suitable parameters to optimize the model’s objective function. Optimization algorithms include Gradient Descent, Newton’s Method, Quasi-Newton Methods, Conjugate Gradient Method, Swarm Intelligence Optimization algorithms. Swarm Intelligence Optimization simulates the behavioral characteristics of biological groups in nature, encompassing Particle Swarm Optimization (PSO), Ant Colony Optimization (ACO), Whale Optimization Algorithm (WOA), Grey Wolf Optimizer (GWO), and others. Research of Al-Dahidi et al. confirms that advanced optimization techniques, such as COA can improve the prediction accuracy of ML models in the field of energy production [20–21]; Zhao et al. used a combined model of GM (1,1) and PSO-optimized quadratic exponential smoothing to predict total automobile sales in China from 2023 to 2030 [22].

Despite significant advancements in NEV sales prediction methods, the prediction accuracy remains relatively low due to the limited duration of available data and significant data variability within the data. This paper aims to analyze the spatiotemporal characteristics of new energy vehicle sales in China, with the goal of exploring the applicability of optimization algorithms to GRU models in predicting city-level sales of NEVs, thereby enhancing the accuracy of sales forecasts for new energy vehicles in Chinese cities.

## Materials and methods

### Data

This paper collects and organizes data on the sales and stock of NEVs and conventional vehicles in China spanning from 2008 to 2024, sourced from the China Statistical Yearbook (https://www.stats.gov.cn/sj/ndsj/). Additionally, city-level sales data for the top 200 cities in sales rankings, from January 2021 to August 2024, are obtained from the WeChat official account “Passenger Car Sales Inquiry” (CYCXLCX).

### Methods

#### BiGRU.

**Fig 1:**
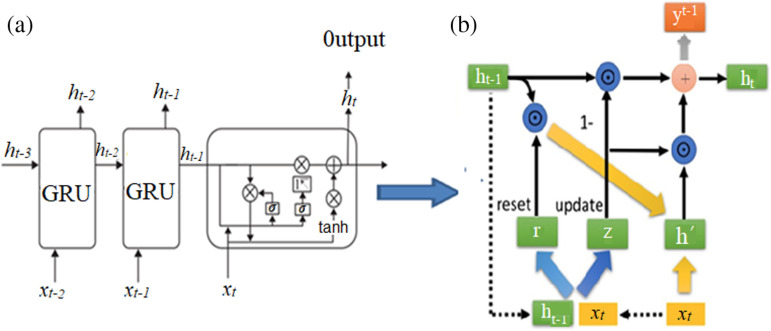
Schematic diagram of GRU network structure.


Long Short-Term Memory (LSTM) is a type of Recurrent Neural Network (RNN) that is particularly suitable for processing and predicting time series data. LSTM resolves the issues of vanishing or exploding gradients in RNNs when dealing with long sequences by introducing three ‘gate’ structures: the forget gate, the input gate, and the output gate [23]. Gated Recurrent Unit (GRU) is a simplified version of LSTM, which couples the output gate and the forget gate into a single update gate, and introduces a reset gate that corresponds to the input gate of LSTM. This simplification reduces the number of parameters and significantly shortens the training time. The schematic diagram of GRU is shown in Fig 1a, while its internal structure is illustrated in Fig 1b [24].

$$r=\sigma \left(W^{r}\frac{x^{t}}{h^{t-1}}\right)$$ (1)

$$z=\sigma \left(W^{z}\frac{x^{t}}{h^{t-1}}\right)$$ (2)

$$h^{t}=\left(1-z\right)⊙h^{t-1}+z⊙h^{\prime }$$ (3)

$$h^{\prime }=\text{tanh}\left(w\frac{x^{t}}{h^{t-1\text{'}}}\right)$$ (4)

where: *σ* represents the sigmoid function, and *W* represents the weight matrix, which involves element-wise multiplication of corresponding elements in the matrices and thus requiring the two multiplied matrices to be of the same shape.

The BiGRU (bidirectional gate recurrent unit) consists of a forward GRU and a backward GRU, capable of processing both the forward and backward sequences of data simultaneously, thereby capturing more feature information.

#### Whale optimization algorithm (WOA).

To improve the prediction accuracy of the GRU model, this paper proposes to use the WOA to optimize the relevant parameters of the GRU model. WOA is a natural inspiration-based optimization algorithm that simulates the behavior of whales, particularly mimicking the ‘bubble-net feeding’ strategy of humpback whales. WOA falls into the category of swarm intelligence algorithms and is widely used to solve continuous and discrete optimization problems. It exhibits strong performance in both global and local search, capable of avoiding local optima and possessing strong global search capabilities [25].

The WOA continuously optimizes solutions by simulating two main strategies employed by whales during predation: the ‘spiral updating behavior’ and the ‘bubble-net behavior’. Specifically, the WOA guides the optimization process by mimicking the behavior of humpback whales as they spiral around prey and form bubble nets for hunting. The WOA algorithm assumes that the current best candidate solution position is the target prey position. After defining the target prey position, other whales attempt to surround and move toward the target prey position. The calculation formula for this process is as follows:


$$D=\left|C\times X*\left(t\right)-X\left(t\right)\right|$$ (5)

Where: *D* represents the distance between the whale and its target (the best food source), *t* denotes the current iteration number, *C* is a random coefficient, $X\left(t\right)$ is the position of the current solution, and $X\text{*}\left(t\right)$is the position of the current optimal solution. Specifically, *C* is calculated as 2r, where r is a random number within the range of [0,1].


$$X\left(t+1\right)=X*\left(t\right)-A\times D$$ (6)

Where: $X\left(t+1\right)$ represents the whale’s position at the next moment, which is updated based on the position of the current best food source and its distance from the food source. *D* denotes the distance between the whale and its target (the best food source), and *A* is a coefficient calculated as A =a * (2r-1). When |A| > 1, the whale may not move directly toward the current best food source but instead conduct a broader exploration within the search space, which helps avoid local optima. When |A| < 1, the algorithm focuses more on refining the current solution, i.e., conducting intensive searches around the current optimal solution. During the iteration process, a gradually decreases from 2 to 0, and the algorithm transitions from global search to local search.

#### WOA-GRU.

**Fig 2:**
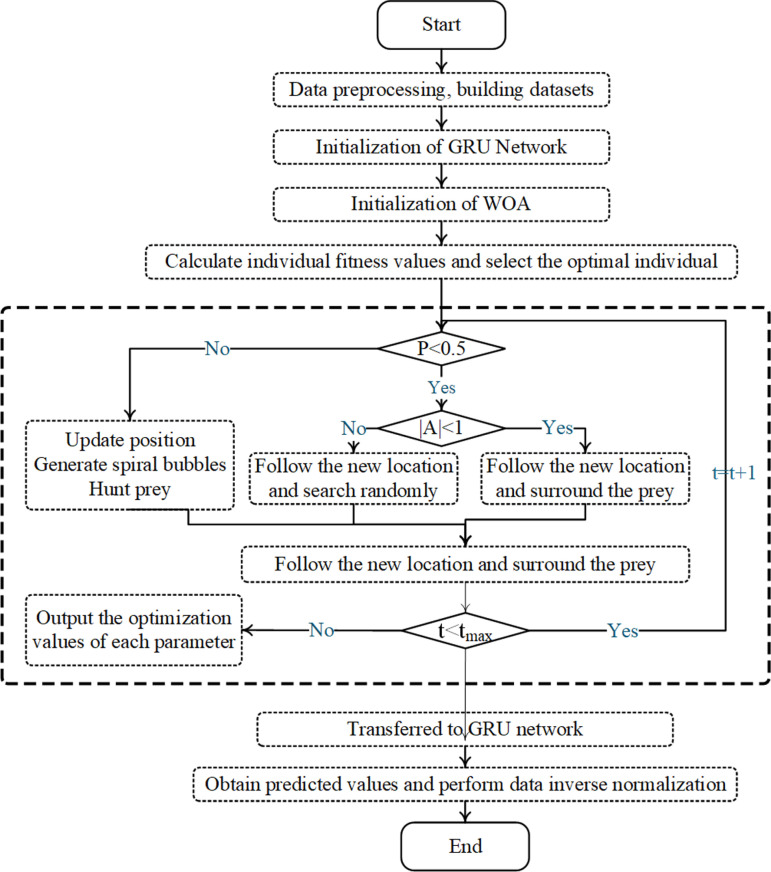
The situation of new energy vehicles in China from 2008 to 2023.

In avoid the pitfalls of speculation and heavy computational burden in the process of parameter tuning, this paper proposes to optimize the relevant parameters of the BiGRU model using the WOA and construct a WOA-GRU model for predicting the monthly sales of NEVs. The construction process of the WOA-GRU model (Fig 2) is described as follows:

## Results

### Spatiotemporal characteristics of the number of NEV in China

Fig 2 illustrates the number of NEVs in China from 2008 to 2023.

As shown in Fig 2, the growth rate of new energy vehicles (NEVs) experienced three significant peaks in the years 2009, 2015, and 2021. Initially, the promotion of electric vehicles (EVs) in China was primarily led by policies, focusing on public sectors such as public transportation, taxis, official duties, municipal services, and postal services. The “Ten Cities, Thousand Vehicles” project jointly promoted by four ministries and commissions in 2009 led to a rapid increase in EV sales, reaching the first peak in 2009. With the implementation of policies such as the exemption of purchase taxes for NEVs in 2014 and the full liberalization of the charging and battery swapping infrastructure market, the sales of NEVs saw growth peaks in 2014 and specifically 2015, with growth rates reaching 325% and 343.5% respectively. As battery, artificial intelligence, and other technologies rapidly developed and NEV technology continued to mature, another small peak in NEV sales growth of 157.5% emerged in 2021. The substantial growth in NEV sales reversed the declining trend in overall automobile sales. After 2017, the growth rate of automobile sales in China slowed significantly to only 3.6%, and even experienced negative growth from 2018 to 2020. It was only in 2021, with the substantial growth in NEV sales reaching 3.52 million vehicles annually, that the negative growth trend in automobile sales was reversed. In terms of the number of NEV sales, the largest increase occurred in 2023, reaching 9.495 million vehicles, compared to 3.52 million and 6.887 million in 2021 and 2022 respectively.

**Fig 3:**
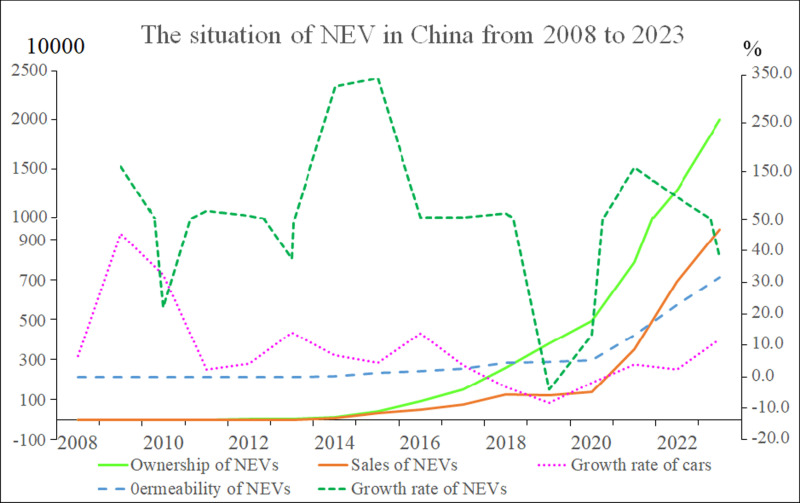
Urban NEV sales in China.

From a city perspective, in 2023, 15 cities including Shanghai, Guangzhou, and Hangzhou had NEV sales exceeding 100,000 vehicles. However, there was a significant disparity in sales among cities. Taking the August 2024 NEV sales data in China as an example, Hangzhou ranked first with 30,529 NEV sales, while Huainan ranked 200th with only 809 sales, a difference of 37.7 times. The disparity in NEV sales among cities is not only significant in quantity but also in spatial distribution. The distribution of the top 200 cities in China in terms of NEV sales in 2023 is shown in Fig 3.

The base map of Fig 3 depicts China’s population density (https://www.resdc.cn/DOI/DOI.aspx?DOIid=33). The Hu Line serves as a dividing line between China’s population development level and economic and social patterns, with 94% of China’s population residing on its eastern side, where the economy is developed. Conversely, only 6% of the population resides on the western side of the Hu Line. As can be seen from Fig 3, the vast majority of the top 200 cities in terms of NEV sales in China are located east of the Hu Line, particularly in the four major economic hubs of the Pearl River Delta, Yangtze River Delta, Beijing-Tianjin-Hebei region, and Chengdu-Chongqing. The sales of NEVs in the northeastern region, which experiences lower temperatures, are relatively low. Similarly, provinces with lower population densities such as Yunnan, Guizhou, and Guangxi also have lower sales of NEVs. The sales of NEVs exhibit a clear spatial distribution pattern.

### Monthly sales forecast of urban NEV based on WOA-BiGRU model

#### Data collection and model training.

This paper utilizes monthly city-level sales data for NEVs from January 2021 to August 2024, a period of rapid growth in China’s new energy vehicle market, focusing on the top 200 cities in sales rankings. After excluding cities with incomplete data over the 43-month period, a total of 7,980 data points were obtained. Each data point includes information on the city, the NEV sales, the year-on-year ratio, the month-on-month ratio, and the penetration rate for that month. As an example, the sales data of the top ten cities in August 2024 are presented in Table 1.

**Fig 4:**
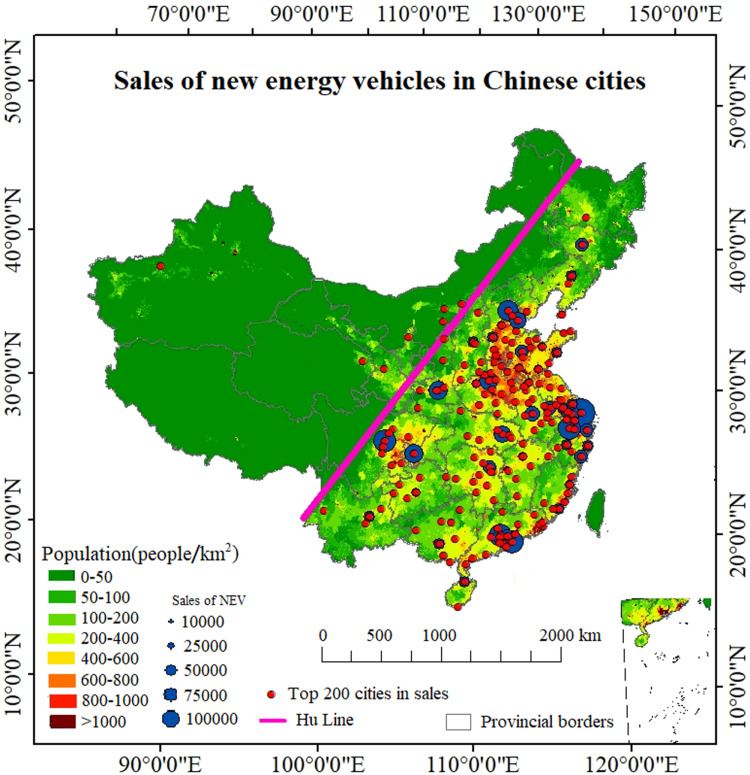
WOA-BiGRU.

The monthly NEV sales model is trained using the constructed WOD-BiGRU model. The BiGRU model consists of an input layer, two hidden layers, and an output layer. This Adam optimizer and Mean Squared Error Loss (MSE Loss) function are employed in this study. Parameters such as the number of neurons in the hidden layers, the number of neurons in the fully connected layer, and the learning rate are optimized using the WOA. The detailed process is illustrated in Fig 4. Before training, the sales data are normalized to mitigate issues caused by large numerical differences and to enhance model performance. To prevent overfitting and improve generalization, a cross-validation method is applied, splitting the training set into a 70% training subset, a 15% validation subset and a 15% test subset.

#### Analysis of prediction results.

**Fig 5:**
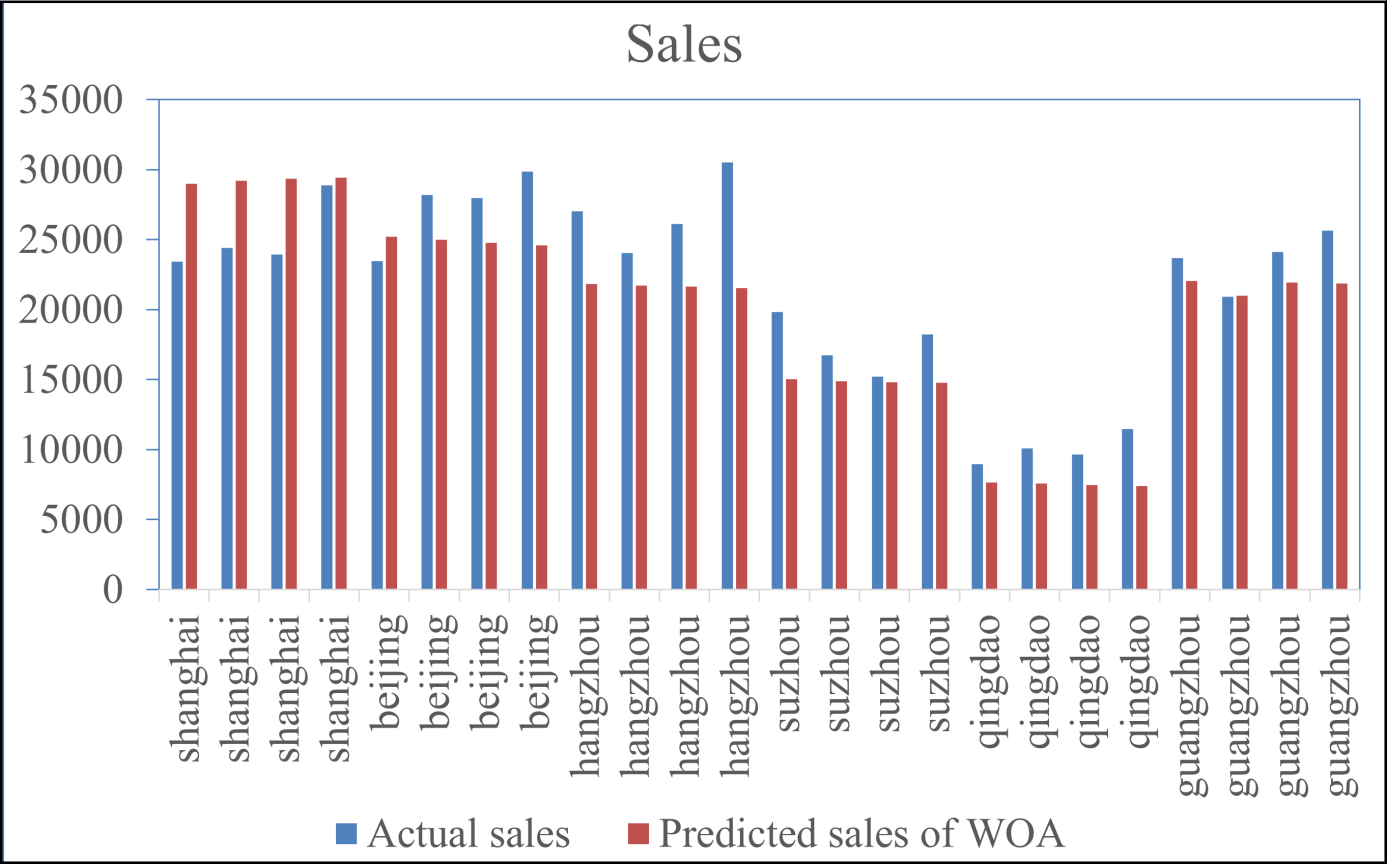
Prediction Results.

Based on the WOA-BiGRU model, the sales data of NEVs in various cities were predicted. The prediction results for cities such as Shanghai, Beijing, Jinan, Suzhou from May to August 2024 as examples, as shown in Fig 5.

To quantitatively analyze the obtained prediction results, the evaluation indices selected were Mean Absolute Error (MAE), Mean Absolute Percentage Error (MAPE), and Root Mean Square Error (RMSE). The MAE, MAPE, and RMSE of this prediction result are as follows: 3051.89, 13.15%, 3784.49, respectively.

#### Analysis of prediction results from different models.

**Fig 6:**
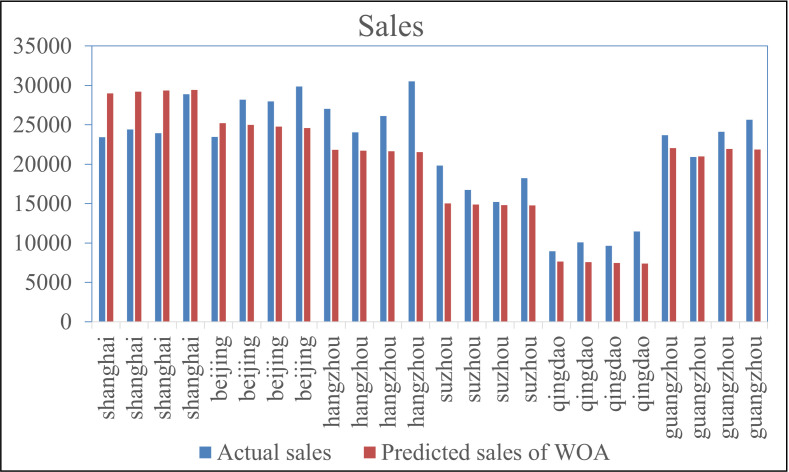
Comparative analysis of prediction results.

To explore the applicability of different optimization algorithms in predicting urban new energy vehicle (NEV) sales, the results of the WOA-BiGRU model were compared with those of the PSO-BiGRU model and the BiGRU model without parameter optimization (Fig 6).

From Fig 6, it can be observed that the predicted sales by both the non-optimized BiGRU and PSO-BiGRU model are slightly lower than the actual sales, while the WOA-BiGRU model are generally higher than the actual sales. The MAE, MAPE, and RMSE of the predictions made by the BiGRU, WOA-GRU, and PSO-BiGRU algorithms are shown in Table 2.

From Table 2, it can be seen that the MAE predicted by the WOA-BiGRU model is 3051.89, which is 526.18 lower than that predicted by the BiGRU model and 104.72 lower than that predicted by the PSO-BiGRU algorithm.

Since 2021, the sales of NEVs in China have entered a phase of explosive growth. Especially in certain cities, where the fluctuations in sales are significant, which has led to lower prediction accuracy in the models.

## Discussion

The sales data of NEVs in cities is more meaningful for the planning of charging infrastructure and power grid regulation. Currently, the publicly available sales data for Chinese cities starts from January 2021. Therefore, the model in this study was constructed using data beginning from January 2021, and its forecasting accuracy is expected to improve as more data accumulates.

**Fig 7:**
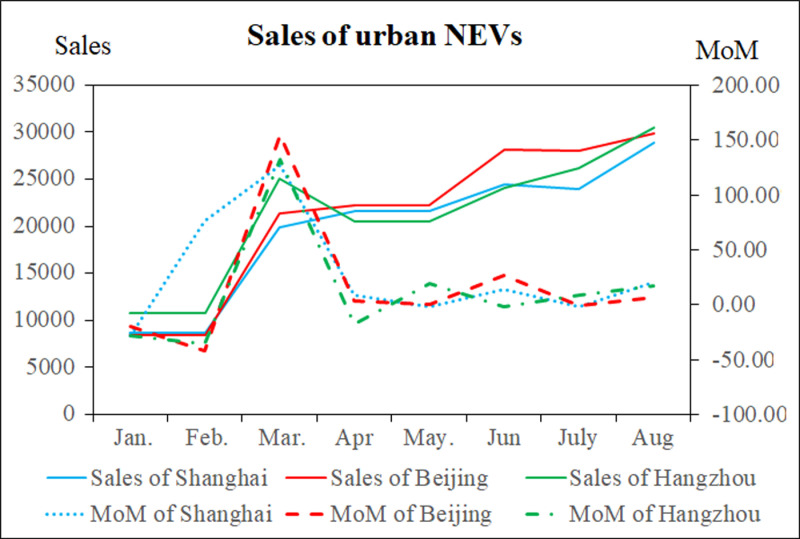
Sales of City New Energy Vehicles.

Although the WOA-BiGRU model developed in this study has achieved good results in predicting urban NEV sales, there are still issues in certain months. For example, in May 2024, the prediction error for NEV sales in Shanghai was as high as 29.1% compared to the actual sales. By examining the raw data, taking Shanghai, Beijing, and Hangzhou (cities with higher sales) as examples, the NEV sales and month-on-month growth rates from January to August 2024 are shown in Fig 7. From Fig 7, it can be observed that Shanghai experienced month-on-month increases of 127.31% and 9.21% in March and April, respectively, but a decrease of -1.57% in May, leading to the predicted sales being higher than the actual sales. In contrast, Beijing saw month-on-month sales decreases of -20.03% and -41.79% in January and February, respectively, followed by increases of 153.87% and 3.78% in March and April, resulting in the model’s predicted sales being lower than the actual sales for those months. In other words, when the trend of sales changes, the prediction accuracy decreases.

Fig 7 also shows that monthly NEV sales in cities fluctuate significantly, thereby further increasing the difficulty of prediction. Urban NEV sales are influenced by a variety of factors, including natural, social, economic, policy, technological, and drivers’ psychology. As previously analyzed, urban NEV sales exhibit significant spatiotemporal variations, with higher sales concentrated in the three major economic centers along the eastern coast and the Chengdu-Chongqing economic center, closely correlated with factors like population density, economic vitality, and ambient temperature. The current WOA-BiGRU model only considers parameters related to NEV sales, such as sales volume, year-on-year growth rate, month-on-month growth rate, and monthly penetration rate. Future research could consider incorporating additional spatiotemporal parameters, such as urban population, economic factors, temperature, as well as technological and policy factors, which could further improve prediction accuracy.

In this study, the optimization of BiGRU model parameters only considered two strategies, namely WOA and PSO, and did not explore or compare other optimization algorithms. A comparative analysis of multiple optimization methods is also a potential direction for future research.

## Conclusion

To achieve precise predictions of urban NEV sales data in China and explore the applicability of optimization algorithms for GRU models in forecasting urban NEV sales., this paper conducted a spatiotemporal analysis of urban NEV sales propose a monthly sales prediction model for urban NEVs based on the WOA-BiGRU algorithm, compared with PSO-BiGRU. The research findings are as follows: There have been three peaks in the growth rate of China’s NEV sales, reversing the declining trend of overall automobile sales in China. There are significant spatiotemporal variations in urban NEV sales, with higher sales primarily concentrated in the four major economic hubs of the Pearl River Delta, Yangtze River Delta, Beijing-Tianjin-Hebei region, and Chengdu-Chongqing. Optimization techniques such as WOA can improve the accuracy of GRU models in predicting city-level sales of NEVs. The WOA-BiGRU model outperforms both the standalone BiGRU and PSO models, achieving a Mean Absolute Error (MAE) of 3051.89, which is 526.18 lower than that of the BiGRU model and 104.72 lower than that of the PSO model.

The WOA algorithm demonstrates better applicability in the field of monthly sales prediction for urban NEVs. This study improves the accuracy of monthly sales predictions for urban NEVs, offering critical insights for the development of the NEV industry in China, the deployment of charging infrastructure, the stabilization of the power grid, and emission reduction in the transportation sector.

**Table 1:** Monthly Sales Data for Cities.

Ranking	Province	Cities	Sales	Month-on-month ratio	Year-over-Year	Penetration rate of month
1	Zhejiang	Hangzhou	30529	16.86%	32.59%	64.25%
2	Beijing	Beijing	29855	6.68%	36.50%	50.25%
3	Shanghai	Shanghai	28885	20.69%	-18.78%	53.74%
4	Guangdong	Shenzhen	26840	-1.86%	30.85%	63.61%
5	Sichuan	Chengdu	26609	3.93%	31.52%	51.23%
6	Guangdong	Guangzhou	25639	6.27%	21.43%	54.42%
7	Chongqing	Chongqing	23849	22.93%	34.85%	60.69%
8	Henan	Zhengzhou	23285	25.28%	19.60%	54.58%
9	Tianjin	Tianjin	21971	15.00%	34.13%	61.03%
10	Hubei	Wuhan	20901	9.30%	42.00%	59.06%

**Table 2:** Comparison of the prediction results.

Model	MAE	MAPE	RMSE
BiGRU	3581.07	15.81%	4370.24
PSO-BiGRU	3156.61	14.02%	3678.47
WOA-BiGRU	3051.89	13.15%	3784.49
